# Multidisciplinary Treatment of Fracture-Related Infection Has a Positive Impact on Clinical Outcome—A Retrospective Case Control Study at a Tertiary Referral Center

**DOI:** 10.3390/antibiotics12020230

**Published:** 2023-01-21

**Authors:** Markus Rupp, Nike Walter, Daniel Popp, Florian Hitzenbichler, Robert Heyd, Sebastian Geis, Melanie Kandulski, Sylvia Thurn, Thomas Betz, Christoph Brochhausen, Volker Alt

**Affiliations:** 1 Department of Trauma Surgery, University Hospital Regensburg, 93053 Regensburg, Germany; 2 Department for Psychosomatic Medicine, University Hospital Regensburg, 93053 Regensburg, Germany; 3 Department of Infection Prevention and Infectious Diseases, University Hospital Regensburg, 93053 Regensburg, Germany; 4 Institute of Clinical Microbiology and Hygiene, University Hospital Regensburg, 93053 Regensburg, Germany; 5 Center of Plastic and Aesthetic, Hand and Reconstructive Surgery, University Hospital Regensburg, 93053 Regensburg, Germany; 6 Department of Internal Medicine I, University Hospital Regensburg, 93053 Regensburg, Germany; 7 Institute of Radiology, University Hospital Regensburg, 93053 Regensburg, Germany; 8 Department of Vascular Surgery, University Hospital Regensburg, 93053 Regensburg, Germany; 9 Institute of Pathology, University Regensburg, 93053 Regensburg, Germany; 10 Institute of Pathology, University Medical Center, 68167 Mannheim, Germany

**Keywords:** multidisciplinary treatment, fracture-related infection, management concept

## Abstract

Objectives: Fracture-related infection (FRI) is a major complication in orthopedic and trauma surgery. The management and choice of treatment can be difficult depending on multiple factors. Therefore, we implemented a weekly multidisciplinary team discussion to determine diagnostic and treatment strategies in FRI patients and aimed to analyze its effect on clinical outcomes. Methods: Clinical outcomes of FRI patients treated before and after implementation of a structured multidisciplinary treatment (MDT) approach with a weekly case discussion were compared at a follow-up of 12 months. Results: In total, *n* = 117 were eligible for enrolment, whereby *n* = 58 patients (72.4% male, mean age 56.7 ± 16.8 years) constituted the MDT group and *n* = 59 patients (72.9% male, mean age 55.0 ± 16.5 years) the control group. In the MDT group more cases were treated with local antibiotics (67.2% vs. 27.1%, *p* < 0.001) and significant less amputations (3.4% vs. 6.8%, *p* = 0.014), as well as less revision surgeries (1.5 ± 1.2 (0–5) vs. 2.2 ± 1.2 (0–7), *p* = 0.048) were performed. A trend towards less debridement, antibiotics and implant retention (DAIR) procedures, lower rates of recurrence of infection and less treatment failures in the MDT group was observable, even though not statistically significant. Conclusion: An MDT approach providing a patient tailored treatment concept in the treatment of FRI patients appears to be beneficial for the affected patients. Quality and efficacy of implemented MDT meetings should further be evaluated to provide sufficient evidence to further implement this valuable tool in clinical practice and decision making.

## 1. Introduction

Fracture-related infection (FRI) is a feared complication after trauma. In recent years the prevalence of FRI has increased [1]. In Germany, 10.5 cases per 100,000 inhabitants were determined in 2019 [2]. In addition, a wide range of infection rates is reported starting from 1–2% after closed fractures, up to 30% after Gustilo–Anderson type III open tibia fractures [3,4]. Mainly, FRIs are caused by *Staphylococcus aureus*, with reported rates of difficult-to-treat pathogens, microorganisms with biofilm-active antibiotic resistance, of about 20–10% [5,6]. Once a biofilm has been established on the implant, the administration of antibiotics alone cannot adequately reduce the bacterial load. Therefore, the therapy of FRI generally involves surgical treatment to control the infection.

A large share of the patients belongs to the elderly population, which often challenges successful treatment [1]. FRI and its treatment have a tremendous impact on the affected patients. Intriguingly, after successful treatment of FRI in terms of infection eradication and bone consolidation, reported quality of life remains reduced even after several years [7]. Moreover, the economic consequences are substantial, whereby healthcare costs range between being 6.5 to 8 times higher than in uncomplicated fracture cases, mainly driven by the prolonged length of hospital stay [8,9,10]. In addition, it was shown that patients developing an infection after fracture had a 45% increase in the odds of receiving social security benefits (odds ratio, 1.45; 95% CI, 1.25–1.68; *p* < 0.001) [11].

To achieve best possible treatment outcomes, several aspects for diagnostics and treatment have to be taken into account: the bone and soft tissue situation, local perfusion, secondary conditions such as diabetes mellitus, advanced age of the patient, antibiotic therapy for several weeks, its potential side effects and not to forget psychological processing of the trauma and FRI as a second hit. These challenges sometimes exceed the expertise of even the well-trained orthopedic and trauma surgeon. Therefore, involvement of experts in the respective field is considered a key element to successful treatment. In oncology, tumor boards have been established and proven to be a useful tool to improve treatment in terms of reduced mortality by discussing cases in a multidisciplinary setting [12,13].

However, in the field of musculoskeletal infection, evidence for successful implementation of a multidisciplinary team (MDT) is still scarce [14]. Research has mainly focused on periprosthetic joint infection (PJI), for which several studies reported a beneficial effect for treatment outcomes implementing an MDT approach [15,16,17]. Improved outcome of tertiary referral centers has been demonstrated for bone infection cases, as well [18].

However, whereas some earlier insights are available in the benefit of an MDT approach for osteomyelitis, no study investigated if an MDT approach is beneficial in the treatment of FRI patients yet [19,20,21]. Therefore, the aim of the present study was to assess influence of implementing a weekly multidisciplinary team meeting to determine diagnostic and treatment strategies for FRI cases on treatment outcomes in terms of mortality, bone consolidation and recurrence of infection and required amputations. The second goal was to assess changes in treatment characteristics after implementation of the weekly multidisciplinary case discussion.

## 2. Results

A total of *n* = 117 patients were eligible for enrolment. Out of these, 58/117 patients (72.4% male, mean age 56.7 ± 16.8 years) were assessed multidisciplinary (MDT), whereas 59/117 patients (72.9% male, mean age 55.0 ± 16.5 years) were treated prior to the establishment of the interdisciplinary meeting. In both groups, FRI mainly occurred at the tibia followed by infections of the ankle and femur. Neither sex, age, BMI, CCI, ASA score, nor anatomical localization and number of initially open fractures differed statistically significant between the groups (Table 1). Methicillin-sensitive *Staphylococcus aureus* (*n* = 46, 39.3%) was the most frequently detected pathogen followed by *Staphylococcus epidermidis* (*n* = 20, 17.1%) [22], whereby there was no statistically significant difference regarding the pathogen distribution between the groups.

Statistically significantly less amputations were performed in the MDT group. Further, more cases were managed with additional local antibiotics (67.2% vs. 27.1%, *p* < 0.001) and less revision surgeries were performed in the MDT group (1.5 ± 1.2 (0–5) vs. 2.2 ± 1.2 (0–7), *p* = 0.048). Length of hospital stay, bone consolidation, and 1-year mortality were comparable in both groups. The overall treatment procedure did not reach statistical significance, whereas a trend towards less debridement, antibiotics and implant retention (DAIR) procedures was observable. Lower rates of recurrence of infection and less treatment failures were found in the MDT group, however not statistically significant (Table 1).

## 3. Discussion

This study compares clinical outcomes of FRI patients with and without a multidisciplinary team diagnostic and treatment approach. The present results demonstrate for the first time a beneficial effect of an MDT approach in patients suffering from FRI in terms of significantly less frequently required limb amputations and surgical revisions such as planned and unplanned re-debridement, whereas one-year mortality, recurrence of infection, bone consolidation were not statistically significantly affected by the MDT case discussions. Surgical strategies did change as well. Less DAIR and one-stage approaches, but more two-stage procedures were performed after implementing the MDT approach. The use of local antibiotics was more often performed after implementation of the weekly multidisciplinary meeting to discuss FRI cases.

In the present study, we assessed different primary outcome measures, which are widely used in clinical research investigating bone and joint infections. For objective parameters such as mortality, bone consolidation and infection eradication, no difference in both investigated treatment groups was determined. However, we found a lower amputation rate in the comparable patient group treated with an MDT approach. This approach entails fruitful discussions starting from reviewing current diagnostics such as lab tests, microbiological and histopathological findings through discussing potential surgical treatment options. For the authors who introduced the weekly interdisciplinary meeting to discuss bone and joint infection cases, this concept seems to be reason for this result. In addition, some evidence exists for MDT to be useful in bone infection and PJI to achieve better results in terms of lower mortality, shorter length of hospital stay, lower reoperation rates due to infection, lower amputation rates and lower overall healthcare utilization [16,18]. The study by Ferguson et al. compared a tertiary referral multidisciplinary bone infection unit with the care in other centers in the rest of England. Our study compared two groups treated at a tertiary care center already specialized in bone and joint infection before and after implementation of a weekly MDT case discussion similar to tumor boards in oncology. Prior to introduction of the MDT meetings, treatment strategies were determined by the treating orthopedic surgeons. Consultations with infectious disease (ID) or other specialists have been performed when ought to be necessary. In other fields such as blood stream infection with *Staphylococcus aureus*, enterococcal bacteriemia or candidemia ID, specialist consultation has been demonstrated to reduce mortality risk significantly [23,24,25]. This may explain minor differences before and after implementation of the MDT infection board. Another reason might be including every FRI case treated in our department in the set time frame. Thus, simple cases were also discussed. In such easier to treat cases, surgical treatment often required no interdisciplinary surgical expertise, whereas completion on diagnostics was checked and antibiotic therapy determined. Pros and cons of often sophisticated surgeries were discussed for more difficult FRI cases requiring for instance revascularization, soft tissue or bone defect reconstruction. Based on these outlined therapy options, joint decisions were consecutively made with the affected patients. On the one hand, this might be reason for non-significant major outcomes such as mortality, bone consolidation and recurrence of infection comparing the historic control group with the MDT group. On the other hand, a lower amputation rate can be explained by the MDT discussions including technical aspects for saving the affected limb. Further, clinically significant differences, albeit not statistically different such as a quarter reduction of treatment failure, seem to be achieved by the MDT approach. Future studies with higher patient numbers might also achieve a statistical difference when comparing different treatment approaches in such heterogenous study groups.

Intriguingly, less surgical revisions have been performed in the MDT-treated FRI cohort compared to the historic control group. Since additional surgical debridement, sometimes simply out of embarrassment or based on formerly common multiple staged surgical treatment concepts, might lead to changes and more difficult to treat microbial patterns, targeted surgical interventions should only be performed in the affected FRI patients [26]. Additional interventions have been demonstrated to result in reduced likelihood of infection control in PJI patients [27]. Thus, defining treatment strategies by a specialized team necessarily result in lesser surgical revisions. The observation that treatment of respective cases by specialists has a beneficial effect on outcome for the patients in different surgical subspecialties [28,29]. Albeit major outcomes did not change as specialist of the department treated patients before and after MDT implementation, current trends in the treatment of FRI also can be observed in the present study. Recently, the use of local antibiotics in FRI has been reported to reduce infection rate [30,31]. As these can reach high concentrations at the target sites, local antibiotics are a also feasible approach to bypassing the unwanted side effects of systemic antibiotics [5]. Antimicrobial-related severe adverse events were reported in 15% of patients treated for bone and joint infections [32]. Additionally, antimicrobials such as gentamicin and vancomycin can be used locally, which should be considered carefully as systemics antibiotics due to the risk of nephrotoxicity. These were shown to achieve coverage rates up to 94% with low resistance rates in FRI [5]. For this, carrier materials already containing antibiotics are commercially available, whereby and individual combination is also an option to be considered [33]. Furthermore, less DAIR procedures performed are in line with recently published worse outcome performing DAIR in FRI [34,35].

In addition, a better patient and indication selection for DAIR in FRI might be reason for the decrease in DAIR cases in the present study [35]. The changes in surgical strategy together with the beneficial use of local antibiotics and optimized non-surgical therapy might be jointly responsible for the improved outcome observed in the present study.

Looking into other disciplines comparing the situation of bone and joint infection care with that earlier in oncology, it can be stated that the introduction of tumor boards in the 1980s has significantly changed the treatment of cancer all over the world. Today, tumor boards are an indispensable part of oncology diagnostics and therapy decision-making. Meanwhile, they are a standard of care, an integral part of clinical practice and often essential for certification of oncology centers. In oncology, the evidence of the apparent benefit was initially difficult to demonstrate. However, time and further studies have shown the benefits of the MDT approach in several oncology subdisciplines [13,36,37,38].

### Limitations

Several limitations should be recognized for the present study. First, the analysis is based on results from a tertiary referral center providing all kinds of subdisciplines, which is often not feasible for other hospitals. Second, the study design compares a historic group with a later-treated MDT group. This longitudinal comparison might be reason for some differences observed in the study and did not allow us to compare the most important outcome measure, patients’ quality of life. Third, as in general for FRI studies, the study cohorts were very heterogenous, which leaves a larger study cohort to be desired. Thus, subgroup analysis would be possible and patient cohorts could be identified, which especially benefit MDT case discussions. Lastly, difficult to analyze upsides and downsides of MDT discussions such as additional costs for meeting resources, saved costs by improved treatment, improved workflows in daily practice due to less separate expert consultations, started research projects and cooperation as well as being a platform for teaching students by involving them in the meeting and case discussions could not be analyzed. Such aspects have high relevance in the academic arena and should also be acknowledged when dealing with this important topic.

## 4. Materials and Methods

Patients treated for FRI aged 18 years or older in a level 1 trauma center in Germany were screened by the international classification of disease (ICD)−10 diagnosis code T84.6 (Infection and inflammatory reaction due to internal fixation device). Afterwards, patients’ medical charts, surgery protocols, laboratory findings as well as microbiological and histopathological reports were screened for inclusion criteria of FRI. Following the 2018 international consensus meeting on musculoskeletal infection [39], FRI was confirmed by the presence of at least one of the following confirmatory criteria: (1) fistula, sinus tract or wound breakdown; (2) purulent drainage or presence of pus during surgery; (3) phenotypically indistinguishable organisms identified by culture from at least two separate deep tissue/implant specimens (including sonication fluid); and (4) histopathological findings (presence of microorganisms in deep tissue specimens or presence of > five PMN/HPF). Patient characteristics (sex, age, BMI, Charlson comorbidity index (CCI) [40], ASA score at the time of surgery) and details of the infection were retrieved. Then, an experimental and a control group were defined. Cases discussed from 15th June 2019 to 31st December 2020 in the MDT meeting (MDT group) constituted the experimental group. The MDT meeting was held on a weekly basis. Members of the MDT were consultants of the disciplines orthopedic surgery, plastic surgery, vascular surgery, infectious diseases, microbiology, radiology, pathology, psychosomatic medicine, geriatric medicine and endocrinology. During these meetings, each case is evaluated from all perspectives, optimal treatment strategies are developed for each individual patient based on collective expertise on documents in the patient’s medical record. Despite comorbidities and risk-factors, special focus is placed on patients’ wishes and expectations, their compliance and contextual factors such as the social environment and individual psychological resources. For the control group, the inclusion period was defined from 1st January 2016 to 31st December 2018 and consisted of cases that were treated prior to the establishment of the MDT meeting. The follow-up period was set to 12 months for both groups. The study was approved by the institutional ethics committee of the University hospital Regensburg, Germany according to the Helsinki Convention (ref. number 20-1681-104). Informed consent was obtained from all subjects involved in the study. The data were processed anonymously.

Patient characteristics (sex, age, BMI, Charlson comorbidity index (CCI) [40], ASA score at the time of surgery) and further details of infections (site of infection, type of implant, pathogens), as well as treatment procedure and clinical outcomes (number of surgeries involving bony debridement, use of local antibiotics, length of hospital stay, signs of recurrence of infection, fracture consolidation) were assessed retrospectively by reviewing electronic medical records. Achieved bone consolidation was determined with an evaluated RUST score >10 [41]. Treatment failure was defined based on the following aspects (1) clinical signs of recurrence of infection based on suggestive and confirmatory criteria for FRI [39]; (2) subsequent surgical intervention for infection after plant procedure; (3) occurrence of FRI-related mortality (by causes such as sepsis); and (4) amputation of the affected limb.

Descriptive and statistical data analysis was performed using IBM SPSS Statistics software (version 27.0, IBM Corp, Armonk, USA). Frequencies were expressed as numbers and percentages. Continuous parameters were presented as means ± standard deviation (SD) and compared by independent Student’s *t*-test. Chi-square test was used for comparison of categorical variables. For all tests, p values < 0.05 were considered statistically significant. Effect sizes were calculated as Cohen’s d.

## 5. Conclusions

An MDT approach providing a patient-tailored treatment concept in the treatment of FRI patients seems to be beneficial for the affected patients. Quality and efficacy of implemented MDT meetings should further be evaluated to provide sufficient evidence to further implement this valuable tool in clinical practice and decision-making.

## Figures and Tables

**Table 1:** Patient characteristics and clinical outcomes of the FRI group managed with a multidisciplinary team (MDT) and control group.

	FRI “MDT”	FRI Control	Statistical Analysis (*p*-Value)	Effect Size (Cohen’s d)
**Patients**	*n* = 58	*n* = 59		
**Sex**			*p* =0.700	−0.45
Male	*n* = 42 (72.4%)	*n* = 43 (72.9%)		
Female	*n* = 16 (27.6%)	*n* = 16 (27.1%)		
Age	56.7± 16.8 years	55.0 ± 16.5 years	*p* = 0.889	−0.099
BMI (kg/m^2^)	28.0 ± 5.4	27.1 ± 5.2	*p* = 0.230	−0.168
CCI	0.7 ± 1.1	0.3 ± 0.83	*p* = 0.763	0.101
ASA score	2.4 ± 0.88	2.2 ± 0.83	*p* = 0.223	0.198
**Anatomical localization**			*p* = 0.071	−0.101
Humerus				
Radius	*n* = 3 (5.2%)	*n* = 5 (8.5%)		
Femur	*n* = 2 (3.4%)	*n* = 4 (6.8%)		
Tibia	*n* = 9 (15.5%)	*n* = 7 (11.9%)		
Ankle	*n* = 27 (46.6%)	*n* = 23 (39.0%)		
Foot	*n* = 10 (17.2%)	*n* = 11 (18.6%)		
	*n* = 7 (12.1%)	*n* = 9 (15.2%)		
Open fractures	*n* = 15 (25.9%)	*n* = 16 (27.1%)	*p* = 0.820	0.141
**Surgical procedure**			*p* = 0.587	0.097
DAIR	*n* = 4 (6.9%)	*n* = 10 (16.9%)		
1-stage	*n* = 19 (32.8%)	*n* = 22 (37.3%)		
2-stage or more	*n* = 33 (56.9%)	*n* = 23 (39.0%)		
Amputation	*n* = 2 (3.4%)	*n* = 4 (6.8%)	*p* = 0.014 *	0.166
Local antibiotics	*n* = 39 (67.2%)	*n* = 16 (27.1%)	*p* < 0.001 *	−0.282
**Clinical Outcomes** LOS	42.3 ± 34.0 days (4−154)	52.2 ± 35.5 days (8–159)	*p* = 0.208	0.23
Revision rate	1.5 ± 1.2 (0–5)	2.2 ± 1.2 (0–7)	*p* = 0.048 *	0.163
Bone consolidation	*n* = 52 (89.7%)	*n* = 50 (84.7%)	*p* = 0.438	0.145
Recurrence of infection within one year	*n* = 12 (20.7%)	*n* = 16 (27.1%)	*p* = 0.238	0.191
1-year mortality	*n* = 2 (3.5%)	*n* = 2 (3.4%)	*p* = 0.899	0.013
Treatment failure	*n* = 14 (24.1%)	*n* = 19 (32.2%)	*p* = 0.184	0.23

MDT = multidisciplinary team; FRI = fracture-related infection; CCI = Charlson comorbidity index; DAIR = debridement, antibiotics and implant retention; LOS = length of stay. * *p* ≤ 0.05.

## Data Availability

The datasets generated and analyzed during the current study are available from the corresponding author upon reasonable request.

## References

[1] Walter N., Rupp M., Lang S., Alt V. (2021). The epidemiology of fracture-related infections in Germany. Sci. Rep..

[2] Walter N., Rupp M., Baertl S., Hinterberger T., Alt V. (2022). Prevalence of psychological comorbidities in bone infection. J. Psychosom. Res..

[3] Ktistakis I., Giannoudi M., Giannoudis P.V. (2014). Infection rates after open tibial fractures: Are they decreasing?. Injury.

[4] Trampuz A., Zimmerli W. (2006). Diagnosis and treatment of infections associated with fracture-fixation devices. Injury.

[5] Baertl S., Walter N., Engelstaedter U., Ehrenschwender M., Hitzenbichler F., Alt V., Rupp M. (2022). What is the most effective empirical antibiotic treatment for early, delayed, and late fracture-related infections?. Antibiotics.

[6] Rupp M., Baertl S., Walter N., Hitzenbichler F., Ehrenschwender M., Alt V. (2021). Is there a difference in microbiological epidemiology and effective empiric antimicrobial therapy comparing fracture-related infection and periprosthetic joint infection? A retrospective comparative study. Antibiotics.

[7] Walter N., Rupp M., Hierl K., Pfeifer C., Kerschbaum M., Hinterberger T., Alt V. (2021). Long-term patient-related quality of life after fracture-related infections of the long bones. Bone Jt. Res..

[8] Metsemakers W.-J., Smeets B., Nijs S., Hoekstra H. (2017). Infection after fracture fixation of the tibia: Analysis of healthcare utilization and related costs. Injury.

[9] Thakore R.V., Greenberg S.E., Shi H., Foxx A.M., Francois E.L., Prablek M.A., Nwosu S.K., Archer K.R., Ehrenfeld J.M., Obremskey W.T. (2015). Surgical site infection in orthopedic trauma: A case-control study evaluating risk factors and cost. J. Clin. Orthop. Trauma.

[10] Iliaens J., Onsea J., Hoekstra H., Nijs S., Peetermans W.E., Metsemakers W.-J. (2021). Fracture-related infection in long bone fractures: A comprehensive analysis of the economic impact and influence on quality of life. Injury.

[11] O’Hara N.N., Mullins C.D., Slobogean G.P., Harris A.D., Kringos D.S., Klazinga N.S. (2021). Association of postoperative infections after fractures with long-term income among adults. JAMA Netw. Open.

[12] Basendowah M., Awlia A.M., Alamoudi H.A., Ali Kanawi H.M., Saleem A., Malibary N., Hijazi H., Alfawaz M., Alzahrani A.H. (2021). Impact of optional multidisciplinary tumor board meeting on the mortality of patients with gastrointestinal cancer: A retrospective observational study. Cancer Rep..

[13] Di Pilla A., Cozzolino M.R., Mannocci A., Carini E., Spina F., Castrini F., Grieco A., Messina R., Damiani G., Specchia M.L. (2022). The impact of tumor boards on breast cancer care: Evidence from a Systematic literature review and meta-analysis. Int. J. Environ. Res. Public Health.

[14] Walter N., Rupp M., Baertl S., Alt V. (2022). The role of multidisciplinary teams in musculoskeletal infection. Bone Jt. Res..

[15] Karczewski D., Winkler T., Renz N., Trampuz A., Lieb E., Perka C., Müller M. (2019). A standardized interdisciplinary algorithm for the treatment of prosthetic joint infections. Bone Jt. J..

[16] Walter N., Rupp M., Baertl S., Ziarko T.P., Hitzenbichler F., Geis S., Brochhausen C., Alt V. (2022). Periprosthetic joint infection: Patients benefit from a multidisciplinary team approach. Bone Jt. Res..

[17] Biddle M., Kennedy J.W., Wright P.M., Ritchie N.D., Meek R.M.D., Rooney B.P. (2021). Improving outcomes in acute and chronic periprosthetic hip and knee joint infection with a multidisciplinary approach. Bone Jt. Open.

[18] Ferguson J., Alexander M., Bruce S., O’Connell M., Beecroft S., McNally M. (2021). A retrospective cohort study comparing clinical outcomes and healthcare resource utilisation in patients undergoing surgery for osteomyelitis in England: A case for reorganising orthopaedic infection services. J. Bone Jt. Infect..

[19] Ziran B.H., Rao N., Hall R.A. (2003). A dedicated team approach enhances outcomes of osteomyelitis treatment. Clin. Orthop. Relat. Res..

[20] Salvana J., Rodner C., Browner B.D., Livingston K., Schreiber J., Pesanti E. (2005). Chronic osteomyelitis: Results obtained by an integrated team approach to management. Connect. Med..

[21] Bose D., Kugan R., Stubbs D., McNally M. (2015). Management of infected nonunion of the long bones by a multidisciplinary team. Bone Jt. J..

[22] Walter N., Baertl S., Engelstaedter U., Ehrenschwender M., Hitzenbichler F., Alt V., Rupp M. (2021). Letter in response to article in journal of infection: “The microbiology of chronic osteomyelitis: Changes over ten years”. J. Infect..

[23] Tholany J., Kobayashi T., Marra A.R., Schweizer M.L., Samuelson R.J., Suzuki H. (2022). Impact of infectious diseases consultation on the outcome of patients with enterococcal bacteremia: A systematic literature review and meta-analysis. Open Forum Infect. Dis..

[24] Vogel M., Schmitz R.P.H., Hagel S., Pletz M.W., Gagelmann N., Scherag A., Schlattmann P., Brunkhorst F.M. (2016). Infectious disease consultation for Staphylococcus aureus bacteremia—A systematic review and meta-analysis. J. Infect..

[25] Kobayashi T., Marra A.R., Schweizer M.L., ten Eyck P., Wu C., Alzunitan M., Salinas J.L., Siegel M., Farmakiotis D., Auwaerter P.G. (2020). Impact of infectious disease consultation in patients with candidemia: A retrospective study, systematic literature review, and meta-analysis. Open Forum Infect. Dis..

[26] Rupp M., Kern S., Weber T., Menges T.D., Schnettler R., Heiß C., Alt V. (2020). Polymicrobial infections and microbial patterns in infected nonunions—A descriptive analysis of 42 cases. BMC Infect. Dis..

[27] Song S.Y., Goodman S.B., Suh G., Finlay A.K., Huddleston J.I., Maloney W.J., Amanatullah D.F. (2018). Surgery before subspecialty referral for periprosthetic knee infection reduces the likelihood of infection control. Clin. Orthop. Relat. Res..

[28] Liang Y., Wu L., Wang X., Ding X., Liang H. (2015). The positive impact of surgeon specialization on survival for gastric cancer patients after surgery with curative intent. Gastric Cancer.

[29] Vernooij F., Heintz P., Witteveen E., van der Graaf Y. (2007). The outcomes of ovarian cancer treatment are better when provided by gynecologic oncologists and in specialized hospitals: A systematic review. Gynecol. Oncol..

[30] Metsemakers W.-J., Fragomen A.T., Moriarty T.F., Morgenstern M., Egol K.A., Zalavras C., Obremskey W.T., Raschke M., McNally M.A. (2020). Evidence-based recommendations for local antimicrobial strategies and dead space management in fracture-related infection. J. Orthop. Trauma.

[31] Sliepen J., Corrigan R.A., Dudareva M., Wouthuyzen-Bakker M., Rentenaar R.J., Atkins B.L., Govaert G.A.M., McNally M.A., IJpma F.F.A. (2022). Does the use of local antibiotics affect clinical outcome of patients with fracture-related infection?. Antibiotics.

[32] Valour F., Karsenty J., Bouaziz A., Ader F., Tod M., Lustig S., Laurent F., Ecochard R., Chidiac C., Ferry T. (2014). Antimicrobial-related severe adverse events during treatment of bone and joint infection due to methicillin-susceptible Staphylococcus aureus. Antimicrob. Agents Chemother..

[33] Walter N., Rupp M., Krückel J., Alt V. (2022). Individual and commercially available antimicrobial coatings for intramedullary nails for the treatment of infected long bone non-unions—A systematic review. Injury.

[34] McNally M., Corrigan R., Sliepen J., Dudareva M., Rentenaar R., IJpma F., Atkins B.L., Wouthuyzen-Bakker M., Govaert G. (2022). What Factors affect outcome in the treatment of fracture-related infection?. Antibiotics.

[35] Buijs M.A.S., van den Kieboom J., Sliepen J., Wever K.L.H., van Breugel J.M., Hietbrink F., IJpma F.F.A., Govaert G.A.M. (2022). Outcome and risk factors for recurrence of early onset fracture-related infections treated with debridement, antibiotics and implant retention: Results of a large retrospective multicentre cohort study. Injury.

[36] Taberna M., Gil Moncayo F., Jané-Salas E., Antonio M., Arribas L., Vilajosana E., Peralvez Torres E., Mesía R. (2020). The multidisciplinary team (mdt) approach and quality of care. Front. Oncol..

[37] Basta Y.L., Baur O.L., van Dieren S., Klinkenbijl J.H.G., Fockens P., Tytgat K.M.A.J. (2016). Is there a Benefit of multidisciplinary cancer team meetings for patients with gastrointestinal malignancies?. Ann. Surg. Oncol..

[38] Pillay B., Wootten A.C., Crowe H., Corcoran N., Tran B., Bowden P., Crowe J., Costello A.J. (2016). The impact of multidisciplinary team meetings on patient assessment, management and outcomes in oncology settings: A systematic review of the literature. Cancer Treat. Rev..

[39] Metsemakers W.J., Morgenstern M., McNally M.A., Moriarty T.F., McFadyen I., Scarborough M., Athanasou N.A., Ochsner P.E., Kuehl R., Raschke M. (2018). Fracture-related infection: A consensus on definition from an international expert group. Injury.

[40] Charlson M.E., Pompei P., Ales K.L., MacKenzie C. (1987). A new method of classifying prognostic comorbidity in longitudinal studies: Development and validation. J. Chronic Dis..

[41] Cooke M.E., Hussein A.I., Lybrand K.E., Wulff A., Simmons E., Choi J.H., Litrenta J., Ricci W.M., Nascone J.W., O’Toole R.V. (2018). Correlation between RUST assessments of fracture healing to structural and biomechanical properties. J. Orthop. Res..

